# Tregitope-linked Refined Allergen Vaccines for Immunotherapy in Cockroach Allergy

**DOI:** 10.1038/s41598-018-33680-9

**Published:** 2018-10-19

**Authors:** Pannathee Prangtaworn, Urai Chaisri, Watee Seesuay, Kodchakorn Mahasongkram, Nattawat Onlamoon, Onrapak Reamtong, Anchalee Tungtrongchitr, Nitaya Indrawattana, Wanpen Chaicumpa, Nitat Sookrung

**Affiliations:** 1 Graduate Program in Immunology, Department of Immunology, Faculty of Medicine Siriraj Hospital, Mahidol University, Bangkok, Thailand; 2 Center of Research Excellence on Therapeutic Proteins and Antibody Engineering, Department of Parasitology, Faculty of Medicine Siriraj Hospital, Mahidol University, Bangkok, Thailand; 3 Department of Tropical Pathology, Faculty of Tropical Medicine, Mahidol University, Bangkok, Thailand; 4 Biomedical Research Incubator Unit, Department of Research, Faculty of Medicine Siriraj Hospital, Mahidol University, Bangkok, Thailand; 5 Department of Tropical Molecular Biology and Genetics, Faculty of Tropical Medicine, Mahidol University, Bangkok, Thailand; 6 Department of Microbiology and Immunology, Faculty of Tropical Medicine, Mahidol University, Bangkok, Thailand

## Abstract

Allergen-specific immunotherapy (AIT) facilitates long-term resolution of allergic morbidity resulting in reduced drug use and increased refractoriness to new sensitization. AIT effectiveness has been demonstrated in seasonal and perennial allergies, and insect stings. However, data and studies in AIT relative to cockroach (CR) allergy are relatively scarce. In this study, mice allergic to American CR (*Periplaneta americana*) were treated with a liposome (L)-entrapped vaccine made of mouse Tregitope289-Per a 9 of the CR, Tregitope167-Per a 9, or Per a 9 alone – or placebo. Allergic mice that received an individual vaccine intranasally had reduced Th2 response, reduced lung inflammation, and reduced respiratory tissue remodeling. However, only L-Tregitope289-Per a 9 and L-Tregitope167-Per a 9 induced expression of immunosuppressive cytokine genes (*IL-10*, *TGF-β*, and *IL-35* for L-Tregitope289-Per a 9, and *IL-10* and *TGF-β* for L-Tregitope167-Per a 9) and increment of idoleamine-2,3-dioxygenase 1 (IDO1), indicating that these vaccines caused allergic disease suppression and reversal of respiratory tissue remodeling via generation of regulatory lymphocytes. Liposome entrapped-recombinant Per a 9 (L-Per a 9) did not cause upregulation of immunosuppressive cytokine genes and IDO1 increment; rather, L-Per a 9 induced high expression of *IFN-γ* in lungs of treated mice, which resulted in mitigation of allergic manifestations. This study provides compelling evidence that both liposome-entrapped vaccines made of single refined major allergen alone and single refined major allergen linked with Tregitopes are effective for reducing allergen-mediated respiratory tissue inflammation and remodeling, but through different mechanisms.

## Introduction

Cockroach (CR) is a pestiferous source of human pathogen and allergen^1,2^. Exposure to CR-derived proteins is associated with high risk of developing allergies, including allergic dermatitis, allergic rhinitis, and atopic asthma^3^. It has been reported that 26–61% of allergic patients have positive skin test to cockroach extract^4–7^. Morbidity caused by CR allergens is usually more severe and prolonged than morbidity caused by other indoor allergens, such as house dust mites (HDM) and pets^8^. After allergen exposure, the sensitized subject develops predominant Th2 immune response^9–11^. Specifically, naïve CD4^+^ T cells transform to Th2 cells, which produce the following Th2 cytokines: interleukin (IL)-4, IL-5, IL-9, and IL-13. The immune response may be aggravated by IL-25, IL-31, and IL-33 produced by activated T cells, alveolar macrophages, epithelial cells, and dendritic cells (DCs)^10–14^. Secreted IL-4 and IL-13 influence class-switching of B cells to secrete an excessive amount of specific IgE that fixes to Fc epsilon receptors (FcεRI) on the surface of tissue mast cells and blood basophils. The sensitized subject also has allergen-specific memory B and Th2 cells, and a high level of serum IgE. Upon re-exposure, the allergen cross-links IgE located on the surface of sensitized mast cells and basophils, causing the cells to release mediators that result in allergic symptoms that can be as severe as atopic asthma or life-threatening immediate anaphylaxis. Asthmatic subjects have chronic airway inflammation, with infiltration of inflammatory cells into respiratory tissue. Persistent inflammation causes tissue remodeling, which is manifested histologically as hyperplasia and hypertrophy of epithelial and goblet cells and excessive production of mucus, thickening of reticular basement membrane, deposition of collagen in the airway wall, subepithelial fibrosis, airway smooth muscle hypertrophy, hyperplasia and degeneration, and angiogenesis^15–21^. These changes limit respiratory air flow, and allergic subjects suffer airway irritability (airway hyper-responsiveness, AHR) and lung function impairment that are not reversible by pharmacologic treatment^20–22^.

Allergen-specific immunotherapy (AIT) has been used to treat allergy since the early 19^th^ century^23,24^. AIT causes long-term (several years) mitigation, if not permanent elimination, of the symptoms, which leads to reduced drug use and increased refractoriness to new sensitizations^25^. AIT effectiveness has been demonstrated for seasonal pollinosis, insect stings, and perennial pet and mite allergies^10,26,27^. AIT regime consists of repeated administrations of small yet increasing amounts (updosing) of the allergen to which the patient is sensitive [either subcutaneously (subcutaneous immunotherapy; SCIT) or sublingually (sublingual immunotherapy; SLIT)] at frequent intervals over a period of time (several months) until a maintenance dose (the dose that the patient can tolerate without developing symptoms) is achieved. The maintenance dose is then given every few weeks or monthly for a few more years. It is believed that AIT has the effect of changing pronounced Th2 response to non-pathogenic Th1 response, and/or effectuating induction of peripheral tolerance by generating regulatory T and/or B cells (Tregs/Bregs) that produce immunosuppressive cytokines (IL-10, TGF-β, and IL-35) and other factors that control effector T cells (Teff) and that increase production of IgG4 and IgA by allergen-specific B cells^28–32^. However, AIT using crude allergenic extracts often causes adverse side effects (especially during the updosing phase) that can range from local reactions (itching, swelling, and/or redness at the vaccine application site, watery eyes, sneezing, rhinorrhea, and/or, stuffy nose) to systemic reactions (urticaria, fever, fatigue, dyspnea, and/or systemic anaphylaxis)^27,33^. In addition, treated subjects may have new IgE response to other components present in the allergenic extract. Thus, a safer and more effective allergen immunotherapy strategy is needed that effectively counteracts the adverse effects of the allergen, but that ameliorates the aforementioned risks and potential adverse outcomes.

Epitopic peptides that stimulate Treg expansion, termed Tregitopes, have been identified on IgG molecules across mammalian isotypes^34^. Tregitope sequences of a particular species are highly conserved. There are 5 Tregitopes (15–26 amino acids) that locate in light and heavy chains of the human IgG including T009 and T029 (both locate in VH), T084 (Vκ), T167 (CH1) and T289 (CH2)^35^. There are also 5 Tregitopes in mouse IgG, *i*.*e*., T29 (Vκ), T54 (Vκ-CH1), T134 and T167 (both locate in CH1) and T289 (CH2). The T289 and T167 bind multiple HLA class-II with higher EpiMatrix scores than the other Tregitopes^35^. Tregitope peptides can stimulate CD4^+^CD25^hi^FoxP3^+^ T cells to secrete IL-10, TGF-β, and monocyte chemoattractant protein-1 (MCP-1)^36^. Human peripheral blood mononuclear cells (hPBMCs) combined with a Tregitope of human IgG (hTregitope) caused expansion of natural Tregs (nTregs) and induction of peripheral/inducible Tregs (pTregs/iTregs) that produce immunosuppressive cytokines^34–36^. Treg cytokines modulate DC activity by down-regulating MHC class-II, CD80, and CD86, and by upregulating immunoglobulin-like transcript-3 (ILT3)^35^. Co-incubation of Tregitopes and antigenic protein with hPBMCs effectuated suppression of immune response to the antigen (*i*.*e*., absence of both Teff proliferation and cytokine secretion)^34^. Co-administration of mouse homologue of hTregitope (mTregitope) with dust mite allergen suppressed immune response to the allergen^34^. In this study, we hypothesized that treatment of allergy using Tregitope-allergen fusion protein would cause pathogenic Th2 response to change to allergen-specific Treg generation, which would result in mitigation of respiratory tissue inflammation. Thus, the aim of this study was to investigate the therapeutic efficacy of liposome-entrapped refined allergen vaccines made of mTregitopes linked as fusion proteins to recombinant Per a 9 (arginine kinase) of American cockroach (*Periplaneta americana*) in a mouse model of CR allergy. The Per a 9 was chosen as the vaccine component because this *P*. *americana* protein is the major allergen that sensitized 100% of the cockroach allergic Thai patients^37^. The therapeutic role of liposome-entrapped native Per a 9 alone was investigated previously^28^.

## Materials and Methods

### Preparation of *P*. *americana* crude extract (CRE)

Wild captured adult *P*. *americana* were verified entomologically before washing with sterile distilled water and maintained at −80 °C until use. The frozen cockroaches were ground in liquid nitrogen into fine pieces. A small volume of phosphate buffered saline (pH 7.4) (PBS) was added, and the mixture was homogenized by sonication and centrifuged at 12,000 × *g* at 4 °C for 20 minutes. The supernatant was filtered through a sterile 0.22 μm membrane, and the protein content of the crude cockroach extract (CRE) was quantified by Bradford assay (Bio-Rad Laboratories, Inc., Hercules, CA, USA). The preparation was maintained in 1-ml aliquots at −80 °C until use.

### Preparation of CRE allergic mice and specimen collection

All animal experiments received official approval from the Animal Care and Use Committee (SiACUC), Faculty of Medicine Siriraj Hospital, Mahidol University, Bangkok, Thailand (COA no. 012/2558). All methods were performed in accordance with the guideline and regulation of the National Research Council of Thailand.

Male BALB/c mice, aged 6–8 weeks, were obtained from the National Laboratory Animal Center, Mahidol University, Salaya, Thailand. The animals were housed in a shoe-box type cage under the following conditions: 12/12 hour light/dark cycle, 23–25 °C, and 55–60% humidity. Feed and water were allowed *ad libitum*. Mice allergic to CRE were prepared as previously described^28,37^. Briefly, each mouse was individually and intraperitoneally sensitized with three doses of CRE (150 µg per dose) in PBS mixed 2:1 (v/v) with alum adjuvant (Pierce; Thermo Fisher Scientific, Inc., Waltham, MA, USA) on days 0, 7, and 14. On day 21, 100 µg of CRE in 20 µl PBS was introduced into the nostrils of each primed mouse (10 µl per nostril). On days 23, 25, and 27, mice were nebulized with 10 mg of CRE in 10 ml PBS for 30 minutes. Sham mice were prepared by injecting them with PBS mixed with alum, challenging them intranasally with 20 µl of PBS, and nebulizing them with 10 ml of PBS using the same dose administration timeline as used for the allergenized mice. On day 28, all mice were bled, and CRE-specific IgE, IgG1, and IgG2a in the sera of each mouse were measured by indirect ELISA^28^. Thereafter, six randomly selected allergenized mice and six sham mice were sacrificed by cervical dislocation performed by a qualified veterinarian and/or scientist, both of whom hold certificates from the National Research Council of Thailand (NRCT) authorizing them to manage and participate in the use of laboratory animals. The diaphragmatic lobe of the right lung of each of the selected mice was used for histopathologic investigations by staining with hematoxylin and eosin (H & E), Periodic Acid-Schiff (PAS), and Masson’s Trichrome (TRI) dyes. The remaining lobes of the right lung were used for measuring the level of indole-2,3-dioxygenase 1 (IDO1) and the left lung was used for cytokine study by quantitative reverse transcription-polymerase chain reaction (qRT-PCR) technique.

### Histopathologic investigations of mouse lung tissues

The diaphragmatic lobe of the right lung of randomly selected mice was fixed with 5% paraformaldehyde and 4% sucrose in PBS for 24 hours. Five-μm thick sections were stained with H & E dyes, PAS (Merck Millipore, Billerica, MA, USA), or TRI (Merck Millipore), and then examined under a light microscope (10 × 40 magnification) (Olympus, Tokyo, Japan) by a pathologist who was blinded to the mouse treatments. H & E stained lung sections were observed for morphology of bronchiolar epithelium, submucosal smooth muscle cells, and inflammatory cells that infiltrated peribronchiolar areas. The degree of lung inflammation was graded using a 0 to 3 scoring system, with 0 indicating no infiltration and 3 indicating the highest degree of lung infiltration. Lung sections stained by PAS were observed for numbers of goblet cells (stained red) in bronchiolar epithelium. A scoring system of 0–3 was used to indicate the relative number of goblet cells, with scores of 0 and 3 reflecting the same variations as described for H & E staining^38,39^. Lung sections stained by TRI were studied for collagen deposition (stained blue) and lung fibrosis, with scores of 0 and 3 reflecting the same variations as described for the two aforementioned staining methods. The histopathology criteria for individual lung scores are shown in Table 1. At least three sections per mouse were observed (10 microscopic fields per section), and all bronchioles observed in each microscopic field were included. Mean (±standard deviation) of the histopathologic grades for each staining method in each group of mice was calculated and compared among groups.

**Table 1:** Grades and histological features of mouse lung sections after staining with (A) hematoxylin and Eosin (H & E) dyes, (B) Periodic Acid-Schiff, and (C) Mannson’s Trichrome.

A	
**Grade**	**Histological features revealed by H & E staining**
0	Bronchiole lined with single layer of ciliated epithelial cells. Submucosa contained single layer of smooth muscle cells with thin layer of connective tissue. Capillaries were lined with single layer of squamous epithelium and no inflammatory cell infiltration into peribronchiolar tissue
1	Thickened bronchiole which lined with ciliated pseudo-stratified columnar epithelial cells; mild degeneration of smooth muscle cells; alveoli showed slightly thickened wall with few inflammatory cells in the interstitial area
2	Hyperplasia of bronchiolar epithelial cells; moderate degeneration of submucosal smooth muscle cells; more inflammatory cell infiltrated into peribronchiolar area than grade 1
3	Bronchiolar epithelial cell hyperplasia and occlusion of the airway by sloughed-off necrotic epithelial cells; intense inflammatory cell infiltration which formed more than 4 layers around bronchioles
**B**	
**Grade**	**Number of goblet cells per bronchiole**
0	No goblet cell was observed
1	Up to 5 goblet cells
2	More than 5 to 20 goblet cells
3	More than 20 goblet cells
**C**	
**Grade**	**Histological features revealed by Mannson’s Trichrome Staining**
0	Negligible collagen deposition at the alveolar septa; thin-walled alveoli
1	One layer deposition of collagen in the alveolar septa with mild fibrotic change
2	Moderate collagen deposition; continuous fibrosis of the alveolar septa
3	Excessive collagen deposition, thickened alveolar septa, compressed alveoli, fibroplasia with confluent fibrotic mass

### Indirect ELISA for measuring CRE-specific IgE, IgG1, and IgG2a in mouse serum samples

CRE-specific- IgE, IgG1 and IgG2a in mouse serum samples were measured by indirect ELISA. Wells of a microtiter ELISA plate (Corning, Inc., Corning, NY, USA) were individually coated with 2 μg CRE in 100 μl carbonate-bicarbonate buffer (pH 9.6) at 37 °C until dry. After washing with PBS containing 0.05% Tween-20 (PBST), all wells were blocked with 1% bovine serum albumin (BSA) in PBS. After discarding the excess blocking reagent by washing, mouse serum (diluted 1:4 for IgE, and 1:100 for IgG1 and IgG2a) was added to 3 blocked wells per sample type and maintained at 37 °C for 1 hour. Rabbit anti-mouse IgE-biotin (SouthernBiotech, Birmingham, AL, USA) diluted 1:1,000, goat anti-mouse IgG1-biotin (SouthernBiotech) diluted 1:5,000, and rat anti-mouse IgG2a-biotin (SouthernBiotech) diluted 1:5,000 was used as a secondary antibody for IgE, IgG1, and IgG2a determination, respectively. Streptavidin-horseradish peroxidase (HRP) conjugate (SouthernBiotech) diluted 1:3,000 and 2,2′-Azino-bis(3-ethylbenzthiazoline-6-sulfonic acid (ABTS) substrate (KPL by SeraCare Life Sciences, Inc., Milford, MA, USA) were used for color development. The enzymatic reaction was stopped by adding 100 μl of 1% SDS in distilled water to all wells. OD_405nm_ of the content of each well was determined against blanks, defined as wells to which PBS was added instead of mouse serum.

### Study of cytokines in mouse lung tissue specimens

Expressions of various cytokines, including *IL-4*, *IL-5*, *IL-13*, *TNF-α*, *IFN-γ*, *IL-12a* (*p35*), *IL-12b* (*p40*), *IL-17a*, *IL-10*, *TGF-β*, and *IL-35*, in lung tissues of experimental mice were determined by qRT-PCR, which was performed using 1-step Brilliant II SYBR Green qRT-PCR Master Mix (Agilent Technologies, Santa Clara, CA, USA), as previously described^28^. The oligonucleotide primers used for gene amplification are listed in Supplementary Table 1. Left lung tissue of each mouse was placed in RNA*later* RNA stabilization reagent (RNA *later*^TM^; QIAGEN GmbH, Hilden, Germany) and maintained at −80 °C until use. Total RNA was extracted from tissue using Total RNA Mini Kit (Geneaid Biotech Ltd, New Taipai City, Taiwan). Complementary DNA (cDNA) was synthesized using RevertAid H Minus Reverse Transcriptase (Fermentas; Thermo Fisher Scientific, Inc.), for later use as a template to quantify cytokine gene mRNA. The PCR mixture contained 1 μl of cDNA and 100 nM each of forward and reverse primers in SYBR Green PCR Master Mix (Applied Biosystems, Inc., Foster City, CA, USA). The reaction was run using Mx3005P QPCR System (Agilent Technologies, Inc.) for 40 cycles. The expression of each target gene was normalized to the housekeeping β-actin gene. MxPro QPCR software for Mx3005P QPCR System (Agilent Technologies, Inc.) was used for data analysis.

### Preparation of recombinant *P. americana* Per a 9, mouse Tregitope289-Per a 9 and Tregitope167-Per a 9 fusion proteins, and mouse Tregitopes 289 and 167 peptides

For preparing recombinant Per a 9, total RNA was isolated from crude cockroach extract (CRE) of adult *P*. *americana* using TRIzol reagent (Invitrogen, Carlsbad, CA, USA). Complementary DNA was synthesized using ReverseAid First Strand cDNA Synthesis Kit (Thermo Fisher Scientific, Inc.), for use as a template for PCR amplification of the Per a 9 coding sequence. The following PCR primers were designed from the GenBank database (accession no. AY563004.1): forward: 5′-CGGGATCCGATGGTGGACGCCGCA-3′, and reverse: 5′-GCAAGCTTGAGCGAGCTCTCCAG-3′. The PCR reaction mixture consisted of 1 µl cDNA, 10 µM of the each primer, 2.5 µl of 25 mM MgCl_2_, 2 µl of 2.5 mM dNTP, 0.2 µl of 5 units/µl DNA polymerase, and 40.3 µl of ultrapure distilled water. The thermal cycles were, as follows: initial denaturation at 94 °C for 5 minutes; 30 cycles of 94 °C for 30 seconds, 50 °C for 30 seconds, and 72 °C for 40 seconds; and, final extension at 72 °C for 7 minutes. The PCR product was verified by DNA sequencing before cloning into pTZ57R/T, subcloning into pET23b^+^ expression vector, and deposition into BL21 (DE3) *E*. *coli*.

To prepare the mouse Tregitope289-Per a 9 (T289-Per a 9) and Tregitope167-Per a 9 (T167-Per a 9) fusion proteins, eight nucleotide sequences (designated T289-S1, T289-S2, T289-S3, and T289-S4, and T167-S1, T167-S2, T167-S3, and T167-S4) were synthesized (Bioneer, Inc., Alameda, CA, USA) (Supplementary Table 2). The S2 and S3 sequences were hybridized at the complementary overlapped oligonucleotides, and polymerized with DNA polymerase. The PCR reaction mixture consisted of 10 µM each of S2 and S3, 0.4 µl of 10 mM dNTP, 4 µl of Phusion Hot Start II High-Fidelity Buffer (Thermo Fisher Scientific, Inc.), 0.2 µl of 5 units/µl Phusion Hot Start II High-Fidelity DNA Polymerase (Thermo Fisher Scientific, Inc.), and 14.6 µl of ultrapure distilled water. The thermal cycles were initial denaturation at 98 °C for 30 seconds; 35 cycles of 98 °C for 5 seconds, 49 °C for 10 seconds, and 72 °C for 5 seconds; and, final extension at 72 °C for 5 minutes. The product of the first reaction (designated S2-S3 assembly) was amplified with DNA polymerase using S1 and S4, which contained oligonucleotides that overlapped with the S2-S3 assembly. The PCR reaction mixture consisted of 1 µl of PCR product (S2-S3 assembly) and 10 µM each of S1 and S4. The other PCR ingredients and thermal cycles were similar to those used to prepare the S2-S3 assembly. Tregitope289 (T289) and Tregitope167 (T167) coding DNA sequences were then synthesized. The protocol used to prepare Tregitopes is shown in Supplementary Fig. 1.

The Tregitope-coding DNA fragments that had overlapping regions with the pET23b^+^ expression vector were inserted individually into the pET23b^+^-*Per a 9* vector upstream of the Per a 9 coding sequence using overlap extension PCR^40^. The hybridized insert was extended using Phusion DNA Polymerase and the vector as a template. The PCR reaction mixture consisted of 13.5 ng of Tregitope-coding DNA, 100 ng of pET23b^+^-*Per a 9* recombinant vector, 0.4 µl of 10 mM dNTP, 4 µl of Phusion Hot Start II High-Fidelity Buffer, 0.2 µl of 5 units/µl Phusion Hot Start II High-Fidelity DNA Polymerase, and 12.7 µl of ultrapure distilled water. The thermal cycles were, as follows: initial denaturation at 98 °C for 30 seconds; 25 cycles of 98 °C for 10 seconds and 72 °C for 70 seconds; and, final extension at 72 °C for 5 minutes. The methylated parental plasmid was eliminated by *Dpn*I restriction enzyme digestion. The pET23b^+^-*T289-Per a 9*, pET23b^+^-*T167-Per a 9*, and pET23b^+^-*Per a 9* recombinant plasmids were transformed into JM109 *E*. *coli*. Appropriately transformed *E*. *coli* colonies were screened by PCR using T7 promoter and T7 terminator primers. Plasmid DNAs were extracted from selected clones and verified by sequencing. Thereafter, the pET23b^+^-*T289-Per a 9*, pET23b^+^-*T167-Per a 9*, and pET23b^+^-*Per a 9* recombinant plasmids were isolated from the JM109 *E*. *coli* clones, and transformed into BL21 (DE3) *E*. *coli*. Appropriately transformed BL21 (DE3) *E*. *coli* colonies were grown under 0.4 mM IPTG induction condition for 6 hours. Individual cultures were centrifuged (12,000 × *g*, 4 °C, 30 minutes), and one gram each of the bacterial pellet was suspended in 10 ml BugBuster^TM^ Protein Extraction Reagent (Novagen by Merck KGaA, Darmstadt, Germany), and then added to mix with 20 μl of Lysonase^TM^ Bioprocessing Reagent (Novagen). The preparation was maintained at room temperature for 20 minutes and then centrifuged (8,000 × *g*, 4 °C, 30 minutes). Each pellet (bacterial inclusion body; IB) was washed twice with Wash-100 solution [100 mM of phosphate buffer (pH 8.0), 5 mM of EDTA, 1 M of NaCl, and 1% v/v of Triton X100], once with Wash-114 solution (50 mM of Tris-HCl, 300 mM of NaCl, and 1% v/v of Triton X-114), once with Wash-Solvent reagent (50 mM of Tris-HCl and 60% v/v of isopropanol), and finally washed with distilled water. After centrifugation (8,000 × *g*, 25 °C, 30 minutes), the IB was dissolved in 10% N-lauroyl sarcosine [50 mM of phosphate (pH 7.4), 300 mM of NaCl, and 10% w/v of N-lauroyl sarcosine sodium salt] and sonicated at 40% amplitude and 0.5 cycles per second on ice for 4 minutes. The preparation was centrifuged (15,000 × *g*, 4 °C, 20 minutes) and the recombinant proteins (T289-Per a 9, T167-Per a 9, and Per a 9) were purified using Ni-NTA resin and verified by LC-MS/MS. Concentrations of the protein preparations were determined by Bradford assay and endotoxin contents were determined using Limulus Amoebocyte Lysate assay test kit (Biolasco, Taiwan) before they were maintained in 1-ml aliquots at −80 °C until use.

Mouse Tregitope289 (T289; EEQFNSTFRSVSELPIMHQ) and Tregitope167 (T167; PAVLQSDLYTLSSSVTVPSS) peptides were synthesized (GenScript).

### Ability of T289, T167, T289-Per a 9, T167-Per a 9, and Per a 9 to generate regulatory T cells (Tregs) from mouse peripheral blood mononuclear cells

Mouse peripheral blood mononuclear cells (mPBMCs) were isolated from citrated blood of normal BALB/c mice using Ficoll density gradient centrifugation. Cells were cultured in 96-well tissue culture plates (10^5^ cells/well) in RPMI-1640 medium containing 10% heat-inactivated fetal calf serum (FCS), 100 units/ml penicillin, and 100 μg/ml streptomycin (complete medium). Cells were added in duplicate with either 1 μg T289 peptide, T167 peptide, 20 μg each of recombinant T289-Per a 9 (contained 1 µg of T289), T167-Per a 9 (contained 1 µg of T167), or Per a 9. PBMCs added with 1 μg tetanus toxoid (TT) (Biofarma, Bundung, Indonesia) served as positive control, and cells in medium alone as negative control. Plates were incubated at 37 °C in 5% CO_2_ atmosphere for 24 hours. Cells were combined with anti-CD16/32 (FcX; BioLegend, Inc., San Diego, CA, USA) and incubated at room temperature in darkness for 15 minutes to block FcγIIa and FcγRIII. Cells were washed with PBS containing 2% FCS, and stained with anti-CD3-FITC, anti-CD4-PerCP (BioLegend, Inc.), anti-CD25-BV421, and anti-CD45RA-PE (BD Biosciences, San Jose, CA, USA). Then, surface-stained cells were washed with PBS containing 2% FCS, and intracellular FoxP3 was stained using anti-FoxP3-Alexa Flour^®^647 (BioLegend, Inc.), according to manufacturer’s instructions. Cells were then combined with 500 μl of PBS and subjected to flow cytometric analysis (BD LSRII; BD Biosciences). Forward (medium) and side (low) scattered lights were used to differentiate lymphocytes from other cells. Percent CD3^+^CD4^+^CD25^hi^FoxP3^+^CD45RA^−^ (Tregs) was analyzed using FlowJo software (BD Biosciences).

### Preparation of liposome-entrapped vaccines

Liposome was prepared, as previously described^28^, and used as the vaccine delivery vehicle and adjuvant. Phosphatidylcholine (PC) (Lipoid AG, Steinhausen, Switzerland), cholesterol (C) (Sigma-Aldrich, Steinheim, Germany), and dimethyldioctadecyl ammoniumbromide (DDAB) (Fluka; Honeywell Research Chemicals, Morris Plains, NJ, USA) were mixed at a ratio of 1:1:2, respectively, and dissolved with dichloromethane. One ml each of lipid stock was rotated in a round-bottom flask until a lipid gel film was formed. Residual moisture was removed by leaving the flask in a laminar flow hood. The preparation was maintained at −20 °C until use.

Vaccine components, including T289-Per a 9, T167-Per a 9, and Per a 9 (1.25 mg) in 500 μl of PBS, were added separately to the lipid gel film in individual flasks and rotated until milky and homogeneous suspensions were obtained. For preparing placebo, 500 μl of PBS was added to the lipid gel. The preparations, designated L-T289-Per a 9, L-T167-Per a 9, and L-Per a 9 vaccines and placebo (L-P), were, thus, prepared.

### Vaccine characterization

Zeta potentials, sizes, and the polydispersity indices of the vaccines and the placebo were determined using Zetasizer NanoZS (Malvern Instruments, Malvern, UK). Percent antigen entrapment into lipid micelles was also determined. The L-T289-Per a 9, L-T167-Per a 9, and L-Per a 9 vaccines were then centrifuged (14,000 × *g*, 4 °C, 20 minutes). The protein content of the supernatants was measured by Bradford assay, and percent antigen entrapment was calculated using the following equation: [(total protein added originally to the lipid gel film – protein found in the supernatant)/(total protein added)] × 100.

### Immunization of CRE-allergic mice

Two weeks after CRE nebulization (D41), allergic mice were divided into four groups of six mice each. The first three groups received intranasal administration of 20 μl each of either the L-T289-Per a 9, L-T167-Per a 9, or L-Per a 9 vaccine (10 μl per nostril). Group 4 mice received intranasal placebo (L-P). Seven booster doses were given to primed mice – one booster dose every other day. One week after the last dose (D62), the mice in each individual group were provoked by nebulization with 10 mg CRE in 10 ml PBS. On the next day (D63), all mice were bled, and serum samples were kept for CRE-specific IgE, IgG1, and IgG2a measurement. All mice in all 4 groups were then sacrificed. The diaphramatic lobe of the right lung was subjected to histologic study, and the remaining lobes of the right lung were homogenized. The left lung was placed in RNA *Later*^TM^ solution (Qiagen GmbH) for analysis of cytokine gene expressions by qRT-PCR. Two independent experiments were performed.

### Determination of indoleamine 2,3 dioxygenase 1 (IDO1) concentrations

The levels of IDO1 that contained in the remaining lobes of the right lungs (without diaphragmatic lobe) of the allergic mice treated with Tregitope-allergen vaccines (L-T289-Per a 9 and L-T167-Per a 9), L-Per a 9, and placebo (L-P) were measured using mouse IDO1 ELISA kit (LifeSpan Biosciences, Inc., Seattle, WA, USA) according to the instruction manual. IDO1 standard solutions and samples were added into individual wells of the supplied microplate pre-coated with IDO1 capture antibody and kept at 37 °C for 1 h. After washing with the wash buffer, working solution of reagent A (a biotin-conjugated detection antibody) was added to bind with the captured IDO1. Unbound detection antibody was washed away after incubation at 37 °C for 1 h. An avidin-horseradish peroxidase (HRP) conjugate (working solution B) was added appropriately. A 3,3′,5,5′-tetramethylbenzidine (TMB) substrate was then added which reacted with the HRP resulting in color development. The reaction was stopped by adding sulfuric acid stop solution. OD of the well content was measured at 450 nm. OD_405nm_ of the unknown sample was compared to a standard curve OD_405nm_ in order to calculate the IDO1 concentration of the sample.

### Statistical analysis

SPSS statistics version 17.0 (SPSS, Inc., Chicago, IL, USA) was used for all data analyses. Antibody levels, histopathologic and cytokine data and IDO1 levels were analyzed using one-way analysis of variance (ANOVA), with post hoc comparison using least significant difference (LSD) and independent *t*-test. Data are presented as number and percentage, mean ± standard deviation, or range. A *p*-value less than 0.05 was regarded as being statistically significant.

## Results

### CRE-allergic mice

**Figure 1:**
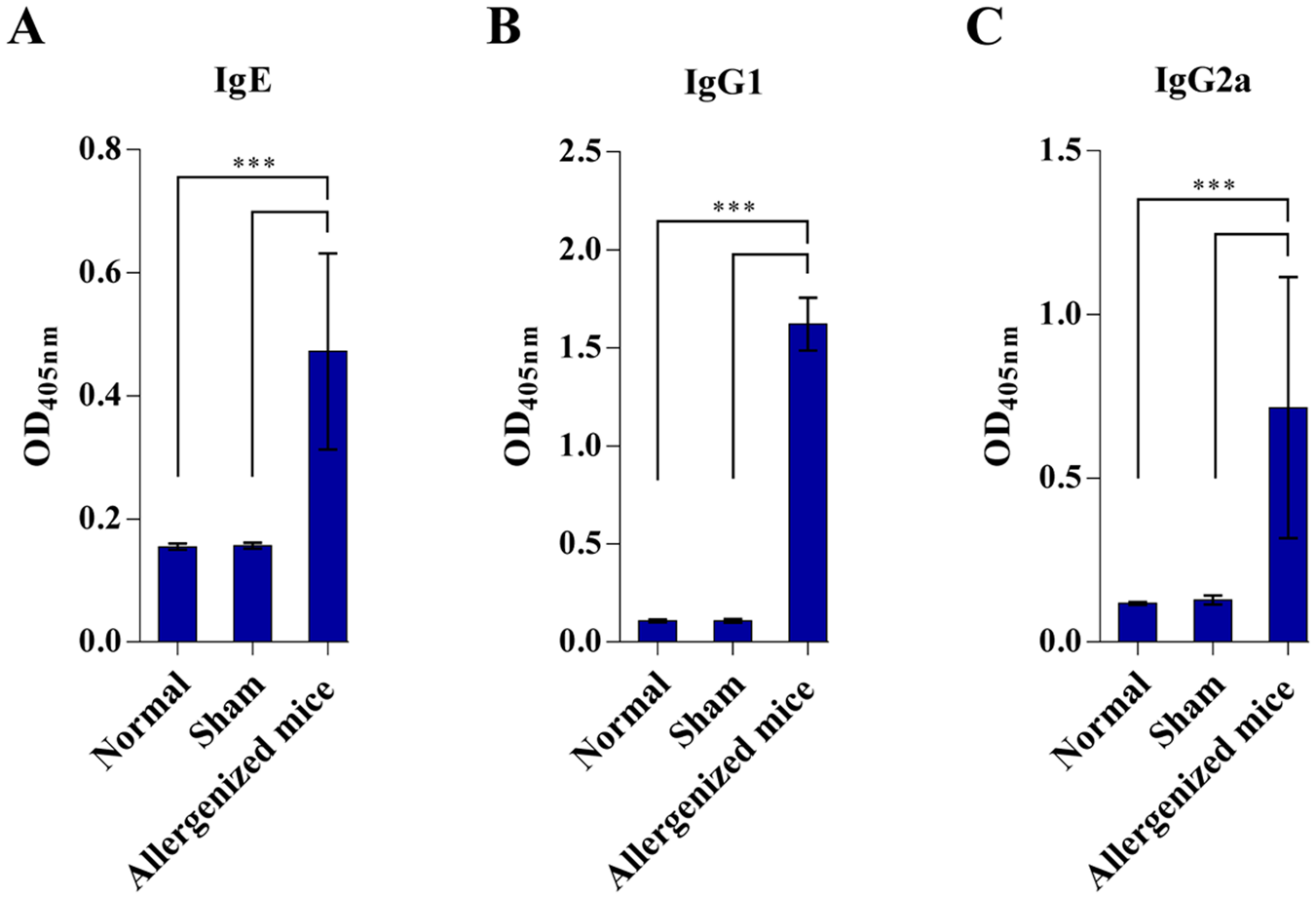
Indirect ELISA for measuring specific IgE, IgG1 and IgG2a in sera of CRE-allergenized mice in comparison to sham and normal mice. (**A**) IgE, (**B**) IgG1, and (**C**) IgG2a.

Results of indirect ELISA for measuring CRE-specific IgE, IgG1, and IgG2a in sera of CRE-allergenized mice compared to sham and normal mice are shown in Fig. 1. Allergenized mice had significantly higher serum IgE (0.472 ± 0.159), IgG1 (1.621 ± 0.135), and IgG2a (0.716 ± 0.399) than both sham mice (0.157 ± 0.005, 0.107 ± 0.010, and 0.128 ± 0.014, respectively) and normal mice (0.155 ± 0.005, 0.107 ± 0.008, and 0.118 ± 0.004, respectively) (*p* < 0.001). Levels of IgE, IgG1, and IgG2a antibodies between sham and normal mice were not significantly different (*p* = 0.983, *p* = 0.999, and *p* = 0.959, respectively).

**Figure 2:**
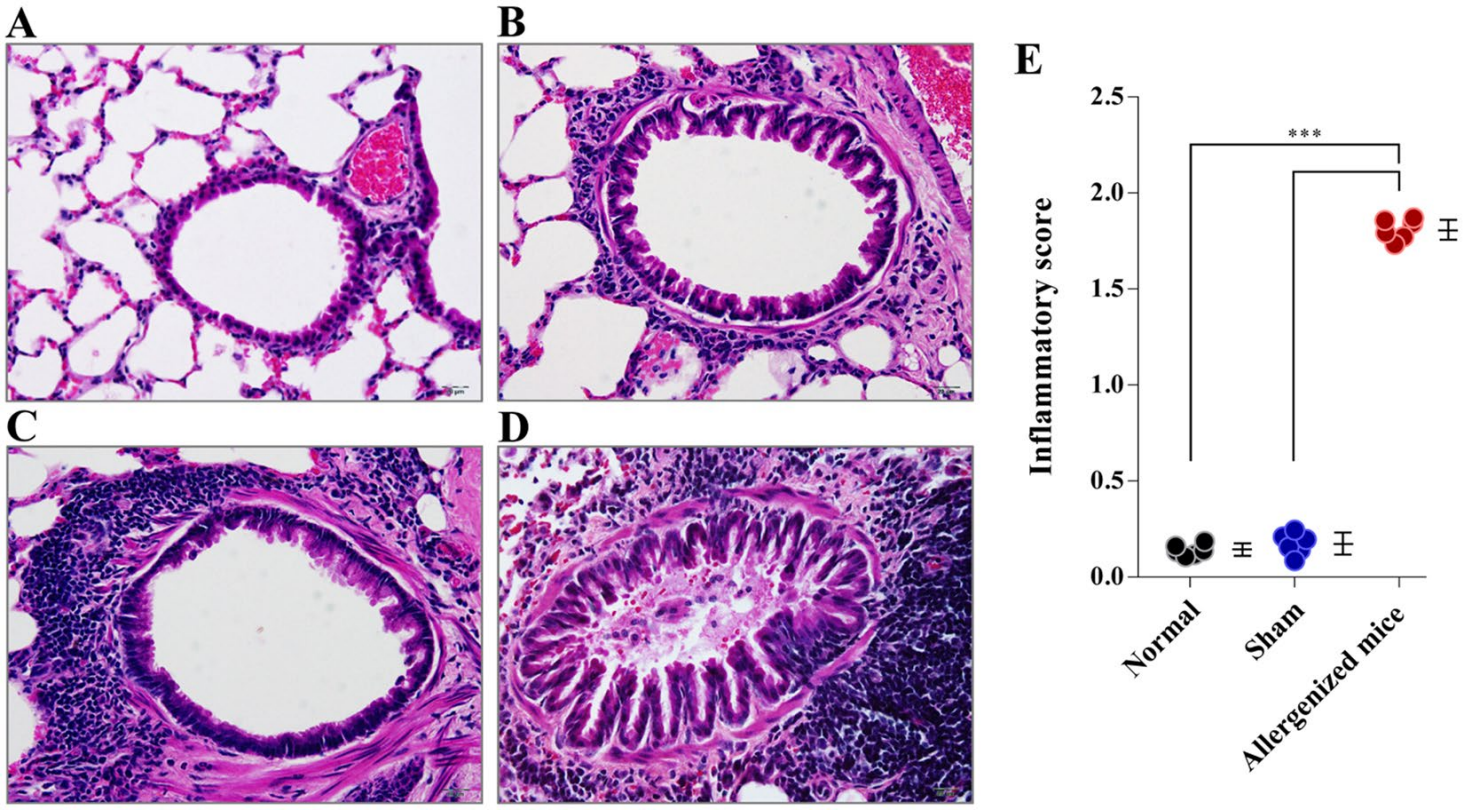
Histological appearances and grades of lung sections of mice after staining with hematoxylin and Eosin (H & E) dyes. (**A**–**D**) Histologic grades 0–3, respectively. (**E**) Comparative average histological grades of normal, sham and allergenized mice. The allergenized mice had significantly higher average lung histopathological grade than the normal and sham mice (*p* < 0.001). The average histologic grades of normal and sham mice were not different (*p* > 0.05).

H & E stained lung sections with different inflammatory grades (grades 0–3) are shown in Fig. 2A–D. The grading criteria are given in Table 1A. CRE-allergenized mice had a mean total lung inflammation score of 1.807 ± 0.481, which was significantly higher than the total scores of sham mice (0.173 ± 0.379) and normal mice (0.143 ± 0.351) (*p* < 0.001) (Fig. 2E).

**Figure 3:**
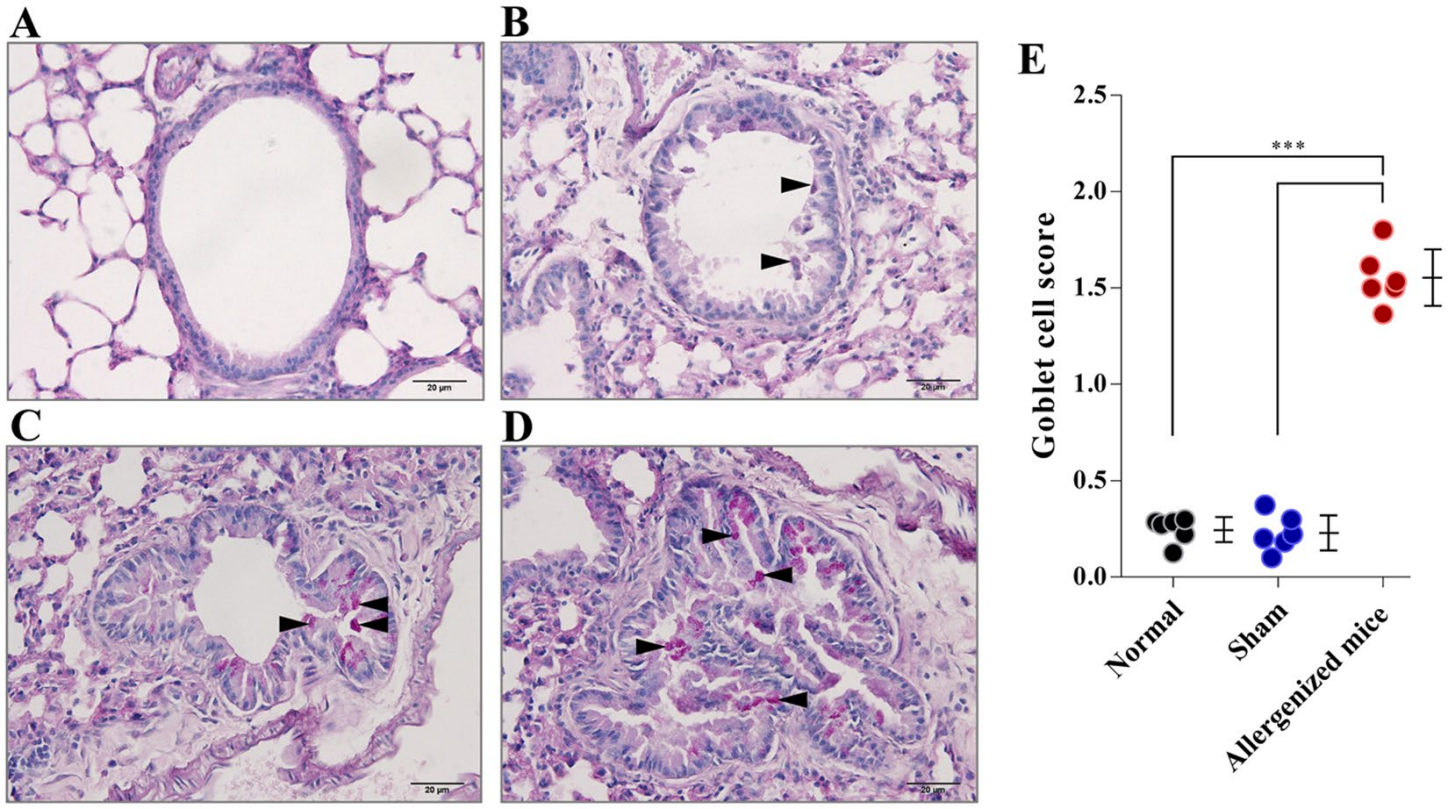
Histological appearances and grades of lung sections of mice after staining with Periodic Acid-Schiff (PAS). (**A**–**D**) Grades 0–3, respectively, of goblet cells (stained pink/red; black arrow heads) in the lung epithelia. (**E**) Comparative average goblet cell grades of normal, sham and allergenized mice. The allergenized mice had significantly higher average goblet cell grade than the normal and sham mice (*p* < 0.001). The average goblet cell grades of normal and sham mice were not different (*p* > 0.05).

Mouse lung sections stained by PAS to illustrate goblet cell grades 0–3 are shown in Fig. 3A–D, and the grading criteria are described in Table 1B. Normal and sham mice had average goblet cell grades of 0.250 ± 0.437 and 0.224 ± 0.421, respectively – both of which were significantly lower than the mean goblet cell grades of allergenized mice (1.551 ± 0.883) (*p* < 0.001).

**Figure 4:**
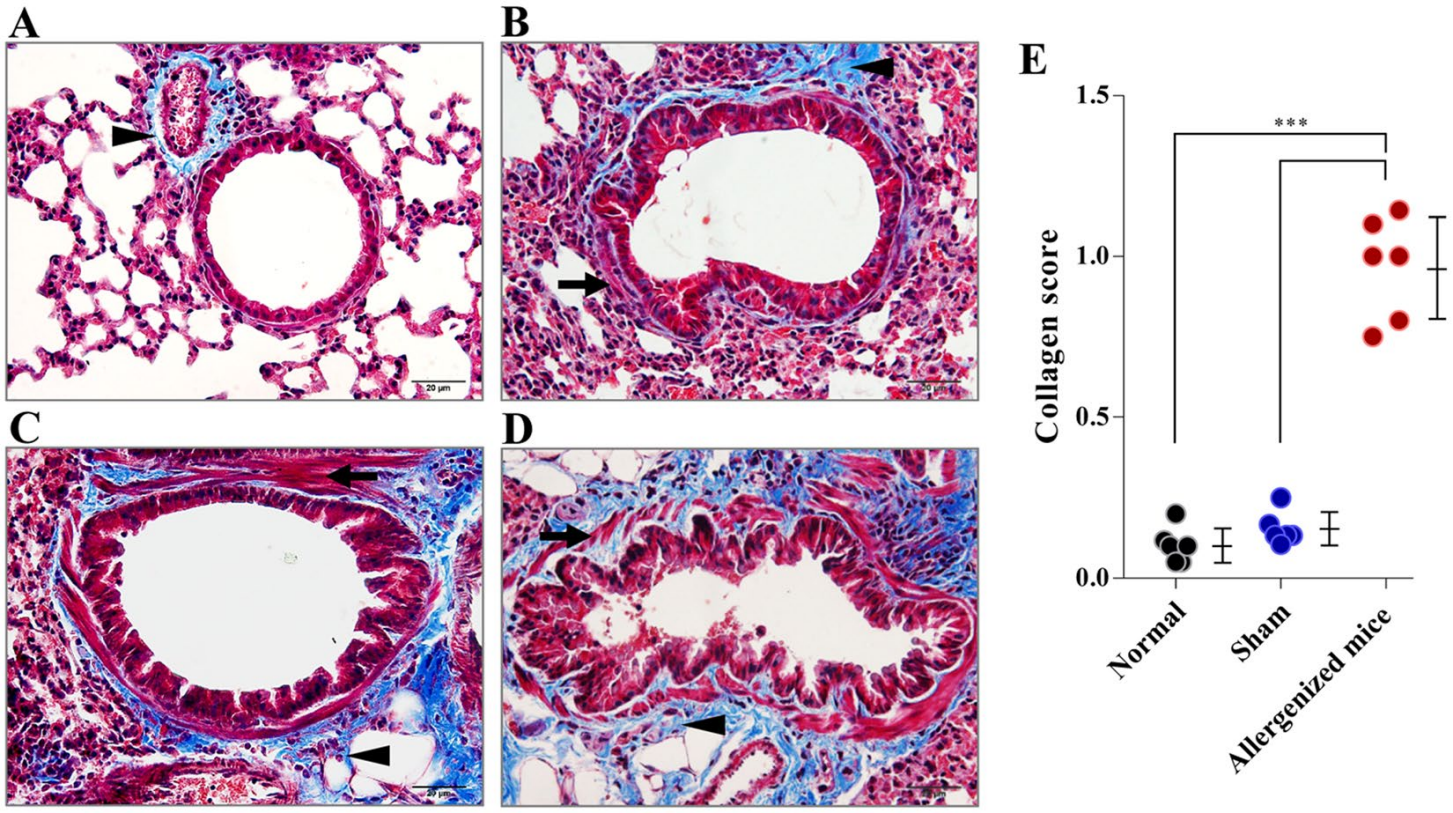
Histological appearances and grades of lung sections of mice after staining with Mannson’s Trichrome (TRI). (**A**–**D**) Grades 0–3, respectively, of collagen (stained blue; black arrow heads) in the bronchiolar subepithelia. (**E**) Comparative average collagen grades of normal, sham and allergenized mice. The allergenized mice had significantly higher average collagen grade than the normal and sham mice (*p* < 0.001). The average collagen grades of normal and sham mice were not different (*p* > 0.05). Black arrows indicate smooth muscle cells.

Figure 4A–D illustrate grades 0–3 of collagen deposition in sub-bronchiolar areas of mouse lungs, with related details given in Table 1C. Allergenized mice had marked increase in collagen deposition around the bronchioles, with an average severity of 0.973 ± 0.552 compared to sham mice (0.160 ± 0.394) and normal mice (0.103 ± 0.306) (*p* < 0.001).

**Figure 5:**
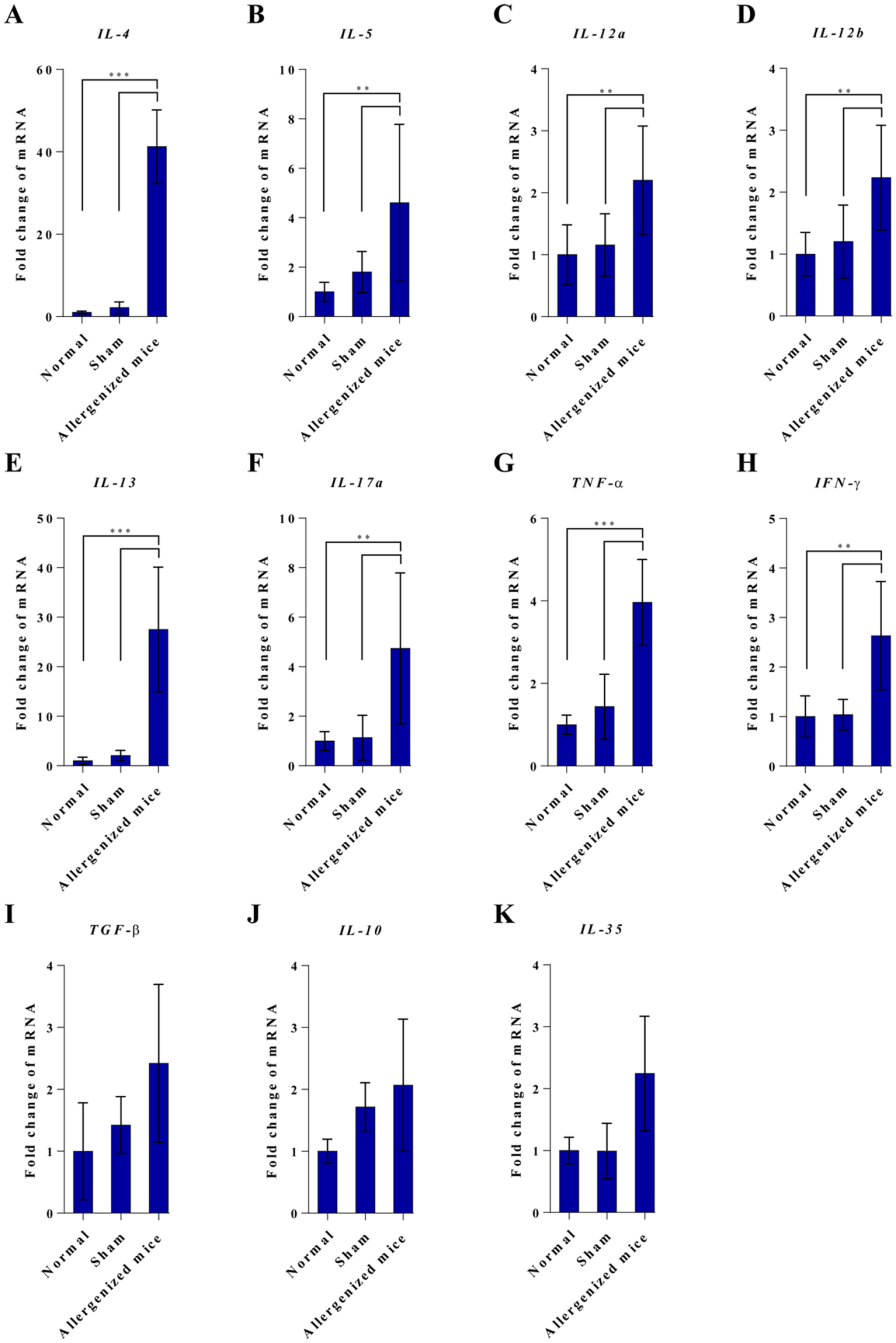
Expressions of cytokine genes in lungs of normal, sham and CRE-allergenized mice. Allergenized mice had up-regulations of Th2 cytokine genes including *IL-4*, *IL-5* and *Il-13*, *IL-12a*, *IL-12b*, *IL-17a*, *TNF-α* and *IFN-γ* compared to normal and sham mice. Expressions of *TGF-β*, *IL-10* and *IL-35* of all mouse groups were not different (*p* > 0.05).

Expressions of cytokines in the lungs of normal, sham, and allergenized mice are shown in Fig. 5. Allergenized mice had significantly higher amounts of *IL-4*, *IL-5*, *IL-12a*, *IL-12b*, *IL-13*, *IL-17a*, *TNF-α*, and *IFN-γ* mRNA than sham and normal mice. Based on CRE-specific IgE levels, lung histopathology, and the cytokine expression profile, the allergenized mice were allergic to the American cockroach. Accordingly, these mice were used to test the therapeutic efficacy of the three liposome-entrapped vaccines.

### Recombinant Tregitope-Per a 9 fusion proteins and Per a 9

Nucleotide sequence coding for T289 and T167 were inserted separately into the pET23b^+^-*Per a 9* vector upstream of the Per a 9 coding sequence using overlap extension PCR, and the recombinant plasmids were introduced into BL21 (DE3) *E*. *coli*. After growing the *E*. *coli* transformants under IPTG induction condition, the hexahistidine-tagged- T289-Per a 9, T167-Per a 9, and Per a 9 were purified from their respective *E*. *coli* lysates using Ni-NTA affinity resin. The recombinant proteins after SDS-PAGE and CBB staining and their respective Western blot patterns are shown in Supplementary Fig. 2A,B, respectively. Endotoxin contents in the recombinant T289-Per a 9, T167-Per a 9, and Per a 9 preparation were 0.20, 0.23, and 0.25 EU/µg, respectively.

### Ability of T289, T167, T289-Per a 9, T167-Per a 9, and Per a 9 to induce Treg generation in mouse PBMCs

**Figure 6:**
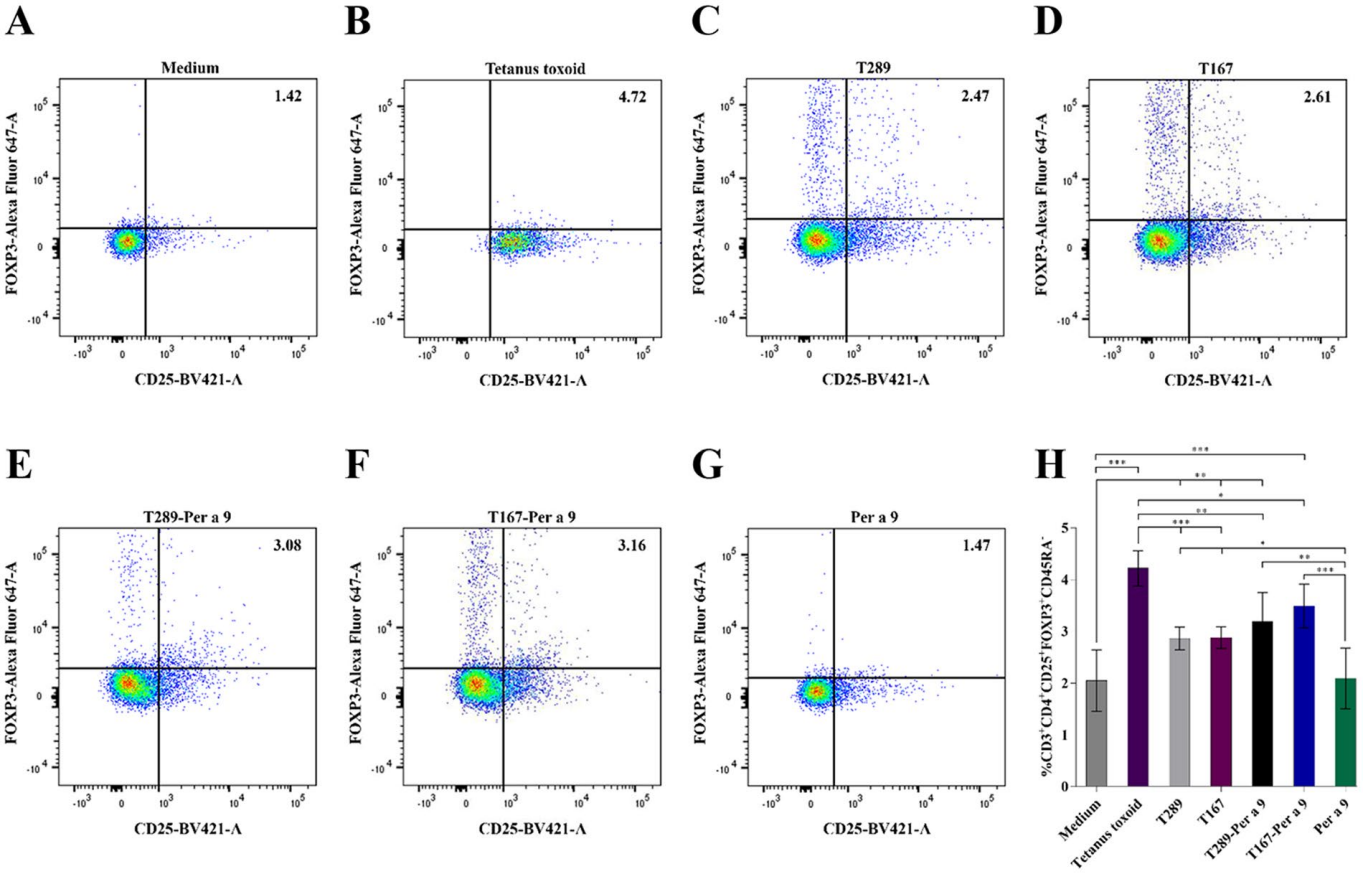
Percentages of CD3^+^CD4^+^CD25^hi^FoxP3^+^CD45RA^−^ T cells (Tregs) in the mouse PBMCs after exposure to: (**A**) medium alone (negative control), (**B**) tetanus toxoid which served as positive control, (**C**) T289, (**D**) T167, (**E**) T289-Per a 9, (**F**) T167-Per a 9, and (**G**) Per a 9 alone, for 24 hours. (**H**) Bar graphs for statistical comparison of the percentages of Tregs in PBMCs after different treatments.

Mouse PBMCs incubated with T289, T167, T289-Per a 9, T167-Per a 9, or Per a 9 alone for 24 hours were stained to identify cells of Treg phenotypes (i.e., CD3^+^CD4^+^CD25^hi^FoxP3^+^CD45RA^−^), after which preparations were subjected to flow cytometric analysis. PBMCs in culture medium alone or incubated with tetanus toxoid were included in the assay as negative and positive Treg generation, respectively. Figure 6 shows the results of flow cytometric analysis of Tregs in mouse PBMCs after various treatments. PBMCs in medium alone contained 1.42% Tregs (Fig. 6A). The percentages of Tregs after treatment with tetanus toxoid, T289, T167, T289-Per a 9, T167-Per a 9, and Per a 9 were 4.72, 2.47, 2.61, 3.08, 3.16, and 1.47%, respectively (Fig. 6B–G). Statistical comparisons of the percentages of Tregs among the treatment groups are shown in Fig. 6H.

### Characteristics of liposome-entrapped vaccines and placebo

Table 2 describes the characteristics of liposome-entrapped T289-Per a 9 (L-T289-Per a 9), L-T167-Per a 9, L- Per a 9, and L-P. The particle size of both the three vaccines and the placebo was larger than 2 μm. The polydispersity indices indicated that the particles in the individual preparations were all fairly homogeneous. Moreover, all of the preparations carried a positive charge. The percentage of immunogen entrapment was more than 90% in all formulations.

**Table 2:** Characteristics of the liposome entrapped CR allergy vaccines and placebo.

Parameter	Vaccine			Placebo L-P
	L-Per a 9	L-T289-Per a 9	L-T167-Per a 9	
Average size (nm) (mean ± SD)	2,168.00 ± 26.00	2,071.67 ± 64.53	5,744.67 ± 340.65	4,519.33 ± 191.11
Polydispersity index (PDI) (mean ± SD)	0.710 ± 0.009	0.565 ± 0.090	0.197 ± 0.180	0.489 ± 0.003
Zeta potential (mV) (mean ± SD)	16.43 ± 0.15	14.47 ± 0.06	26.40 ± 0.36	26.73 ± 0.12
Immunogen entrapment (%)	90.41	91.08	91.90	Not applicable

### Therapeutic efficacy of liposome-entrapped vaccines

**Figure 7:**
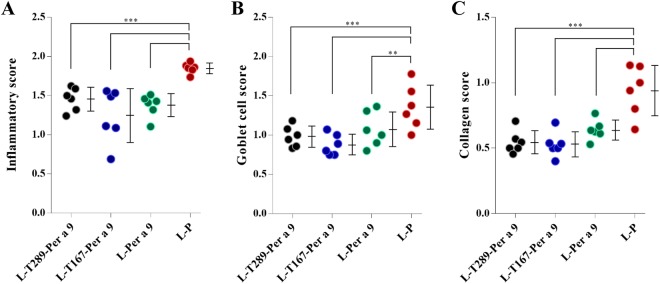
Lung histopathological grades of the CR-allergic mice after immunotherapy with the L-T289-Per a 9, L-T167-Per a 9 and L-Per a 9 vaccines compared to placebo (L-P). (**A**) Grades of inflammatory cells that infiltrated into peribronchiolar areas, (**B**) Grades of goblet cells in the bronchiolar epithelia, and (**C**) Grades of collagen deposition around the bronchioles.

Levels of serum-specific IgE, IgG1, and IgG2 in allergic mice treated with one of the three vaccines or placebo were not different among groups after treatment (Supplementary Fig. 3). In contrast, lung histopathology, including inflammatory grade, number of goblet cells, and collagen deposition around the bronchioles of allergic mice treated with a vaccine, was markedly reduced compared to allergic mice treated with placebo (Fig. 7).

**Figure 8:**
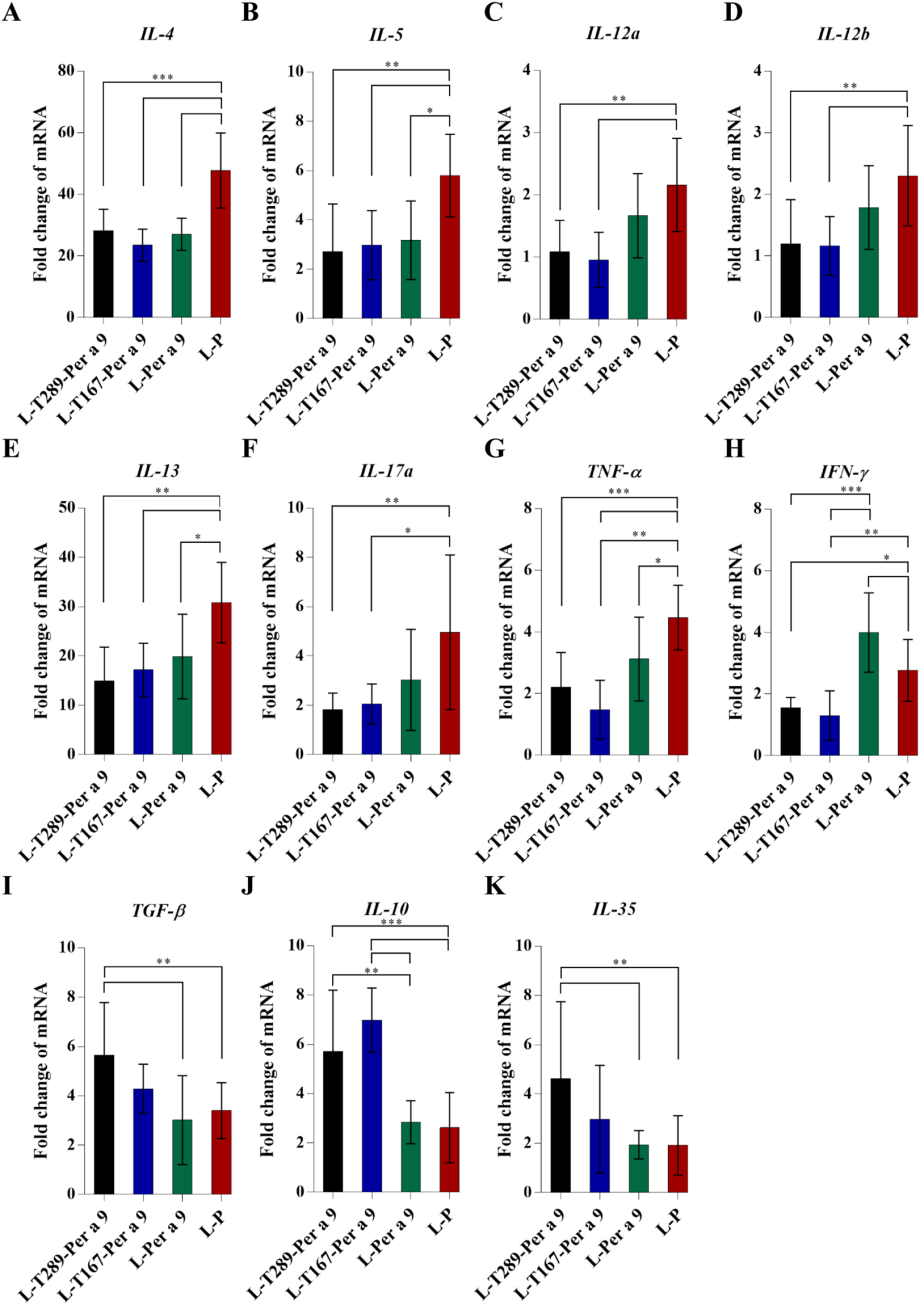
Cytokine expression profiles of the CRE-allergic mice treated with 8 doses of the L-T289-Per a 9, L-T167-Per a 9 and L-Per a 9 vaccines compared to placebo (L-P).

Cytokine expression profiles of allergic mice treated with 8 doses of L-T289-Per a 9, L-T167-Per a 9, L-Per a 9, or placebo (L-P) are shown in Fig. 8. Vaccinated allergic mice had much less *IL-4*, *IL-5*, *IL-13*, *IL-17a*, and *TNF-α* mRNA compared to that of placebo mice. L-T289-Per a 9 and L-T167-Per a 9 caused reduction of *IL-12* and *IFN-γ* mRNA, and upregulation of *TGF-β* and *IL-10* in vaccinated allergic mice compared to placebo mice. Only the L-T289-Per a 9 induced higher expression of *IL-35* in vaccinated mice than in placebo mice. L-Per a 9 could not upregulate expressions of immunosuppressive cytokines in vaccinated mice compared with placebo mice. Allergic mice treated with the L-Per a 9 had higher *IFN-γ* expression than allergic mice treated with the L-T289-Per a 9, L-T167-Per a 9 and placebo mice. The expressions of *IL-12a* and *IL-12b* in the vaccinated mouse groups were not significantly different.

**Figure 9:**
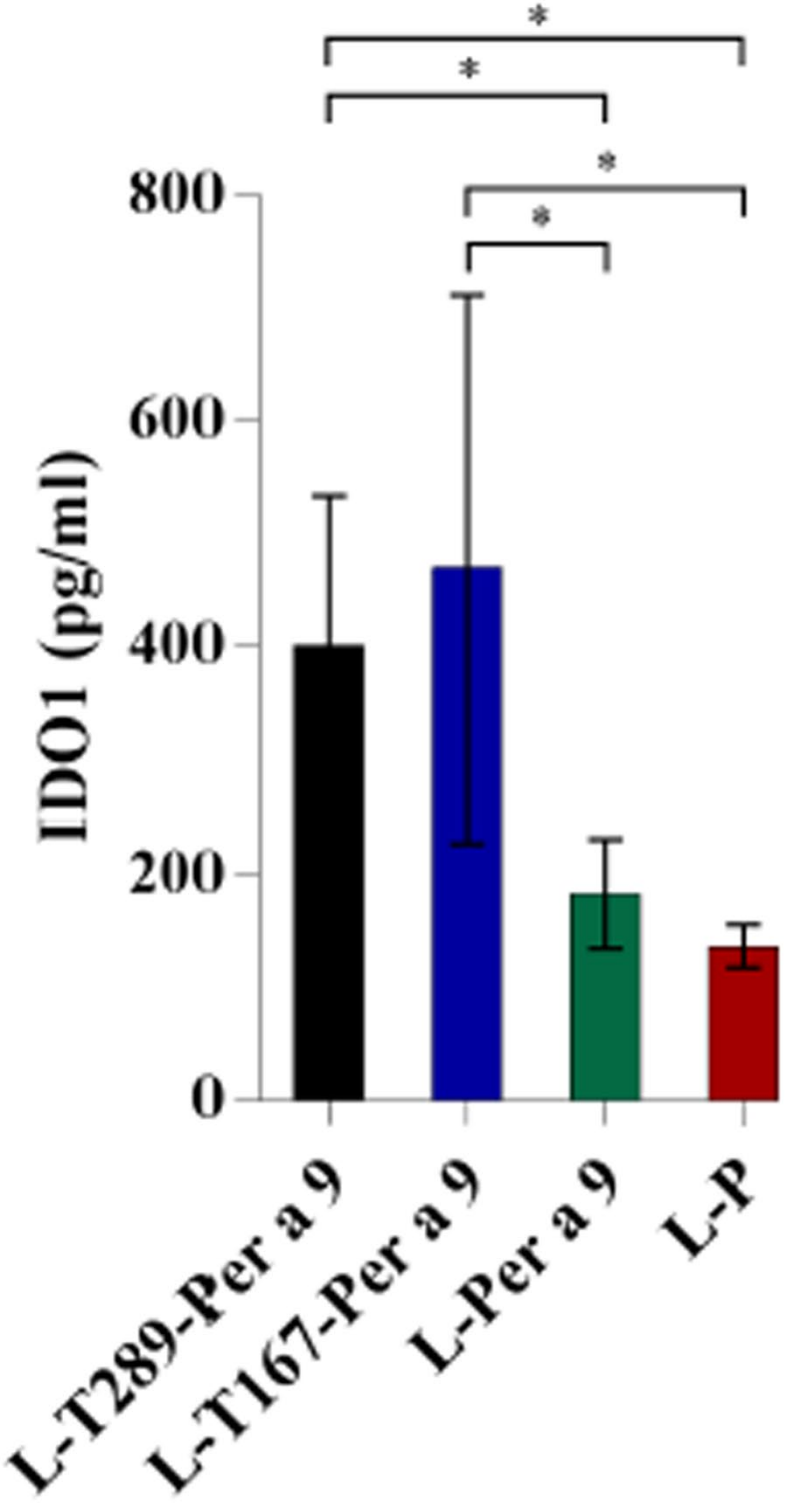
Levels of idoleamine-2,3-dioxygenase (IDO1) in lung tissues of allergic mice treated with T167-Per a 9, T289-Per a 9, and Per a 9 in comparison to the placebo mice. Increment of the IDO was found only in the tissues of the mouse groups treated with Tregitope-allergen fusion proteins. *Indicate significant difference (*p* < 0.05).

Levels of IDO1 in the lung tissues of allergic mice treated with the Tregitope-Per a 9 fusion proteins were increased compared to the placebo mice (Fig. 9). L-Per a 9 did not cause increment of the IDO1 (Fig. 9).

## Discussion

Chronic allergic diseases adversely affect quality of life, with outcomes that range from bothersome symptoms that impair work, learning, and outdoor activities to asthma attacks that not only require emergency treatment, but that may be fatal if not treated promptly and properly^27^. Although current AIT regimes (both SCIT and SLIT) have shown high effectiveness for treatment of many allergies, the data relating to immunotherapy in cockroach allergy are relatively scarce. Cockroaches, particularly *P*. *americana*, are an important indoor allergen source that causes allergic rhinitis and asthma among Asians^41^. Mitigation of CR allergen exposure via pest control and/or health education is difficult and insufficiently effective. Therefore, an innovative vaccine for effective immunotherapy in CR allergy is needed.

In this study, a mouse model of *P*. *americana* allergy was generated using a previously established protocol^28,37^. BALB/c mice were used, given their innate propensity to develop Th2 response upon allergen/antigen stimulation due to their inherently robust production of IL-4^42^. CRE mixed with alum (a Th2 adjuvant) was injected intraperitoneally for priming of naïve mice. The intraperitoneal route was used, as it has been shown that antigen-presenting cells (APCs) in the mouse peritoneal cavity are constitutively mature (high expressions of CD40 and B7 costimulatory molecules, as well as MHC class-II) compared to cells in the respiratory tract^43^. The primed mice were then challenged with the allergen via the respiratory route. The average histopathologic grades of respiratory tissues of experimental mice after completion of allergen sensitization and challenge were significantly higher than those of sham and normal mice, including numbers of inflammatory cells infiltrated into peribronchiolar areas, hypertrophy and hyperplasia of bronchiolar epithelial cells, mucin-producing goblet cells and subepithelial smooth muscle hypertrophy, degeneration and detachment from the basement membrane, and collagen deposition that caused airway wall thickening. All of these histologic features are signatures of respiratory inflammation and tissue remodeling. Moreover, allergenized mice had hallmark signs of Th2 immune response, including increased expressions of Th2 and pro-inflammatory cytokines (the cytokine proteins *per se* and the lung function were not measured), and high serum specific IgE compared to control mice. Thus, the mice that we allergenized were used for further evaluation of the therapeutic efficacy of our liposome-entrapped vaccines.

Various strategies and protocols to increase treatment efficacy, and to reduce the adverse effects of crude allergenic extracts used for AIT have been tested. These strategies include molecular modification of vaccine allergens, altered therapeutic formulations, optimization of the route of vaccine administration, induction of IgE-competitive IgG by means of innovative B-cell-focusing, the use of different types of vaccine carriers and adjuvants, and combined treatment of the allergic entity using both AIT and pharmacologic/biologic agent^44^. In this study, a novel approach is proposed that uses cationic liposome as the vaccine delivery vehicle and adjuvant, a new intranasal route of vaccine administration, and refined Tregitope-recombinant CR major allergen fusion proteins as the vaccine components. Liposome was chosen, as it is known to reduce toxicity/adverse activity of encapsulated components^45^, which in this study were potent CR allergens. The micelles were made of phosphatidylcholine and cholesterol, which are normal constituents of mammalian cell membrane. Allergic mice that received the liposome-entrapped components did not show any sign of morbidity throughout the course of treatment and they all had normal activity and appetite (data not shown). It can, therefore, be inferred that the liposomal vaccines that we produced are innocuous in mouse model.

The size of liposome-encapsulated vaccine and placebo particles was larger than 2 μm, so they should attach readily to and/or be phagocytosed by local APCs^46^. The lipid micelles carried a positive charge; thus, they should coalesce well with the membrane of APCs of nasal-associated lymphoid tissue (NALT), and a large amount of their content could be delivered directly into the cytosol where the processed antigenic peptides should be presented consequently via MHC class-I for CD8^+^ T cell stimulation^47,48^. Even though some of the liposomal vesicles were phagocytosed by APCs, they should be retained in the early endosome where the vaccine component is processed and presented for Th1 response^49^. In both instances, the immune response of vaccinated allergic mice should be deviated away from a deleterious Th2 response. It has been shown that vaccination with recombinant mite allergen complexed with cationic lipid prevents allergic responses to house dust mite^50^. Thus, liposome is well-suited as a delivery vehicle and adjuvant of allergen vaccines that aim to counteract pathogenic Th2 response^51^. The non-invasive and relatively immunogen-sparing intranasal route was selected instead of the sublingual route for vaccine administration. It is expected that immune responses induced by vaccines in upper airway lymphoid tissue can be effective in lower respiratory tissues, which is the affected site of allergen-induced inflammation and remodeling due to the common mucosal traffic of vaccine-stimulated effector cells^52^.

The Tregitopes alone (T289, T167), the T286-Per a 9 and T167-Per a 9 fusion proteins and the recombinant Per a 9 were first tested to observe whether they could generate CD3^+^CD4^+^CD25^+^FoxP3^+^CD45RA^−^ Tregs from cultured mouse PBMCs. The results showed that the Tregitopes and the Tregitope-Per a 9 fusion proteins induced Treg generation which verified the previous notations^34^. The recombinant Per a 9 alone could not induce Treg generation *in vitro*. Because previous study demonstrated that liposome-entrapped native Per a 9 purified from *P*. *americana* extract could mitigate lung inflammation in mice allergic to *P*. *americana* extract^28^, the previous data enticed us to test liposome-entrapped-recombinant Per a 9 vaccine further for therapeutic efficacy, as compared to the L-T289-Per a 9 and L-T167-Per a 9 vaccines and placebo. Although the Tregitopes (T289 and T167) alone could induce Treg generation, they were not included in the subsequent allergic mice treatment experiments. This was not only to minimize the number of animals, but also it is known that generation of inducible Tregs is antigen specific although the immunosuppressive factors secreted/expressed by the so-generated cells (such as IL-10, TGF-β, adenosine) may act non-specifically^53^. We envision that once the allergen-specific Tregs have been generated in the allergic patients by the Tregitope-allergen fusion protein(s), re-exposure naturally to the homologous allergen (or cross-reactive allergen) would act as a booster for reactivation or recall of the allergen-specific memory Tregs^54^ to exert the (higher) immunosuppressive activities. On the other hand, allergen re-exposure should not be able to boost or recall the memory Tregs that had been generated by the Tregitope alone; which means that in order to maintain the respiratory tolerance generated by the Tregitope, the Tregitope must be re-administered to the patients at regular (unknown) intervals, which should not be practical.

Serum CRE-specific IgE levels of all allergic mice treated with a vaccine were not different from before treatment or from the placebo mice that received L-P, which indicated that the humoral responses may have been in the hyperimmune state and sustained until the time of the vaccine efficacy evaluation. It was shown previously that shortly after successful immunotherapy, the level of specific IgE tended to rise and then declined^55^, although the reduced level was not necessarily below the pretreatment level^55^. The role of specific IgG in allergy remains controversial and is still being debated. IgG may be an allergen competitor of IgE in sensitized mast cells/basophils (blocking antibody)^55^. The IgG may cause either cellular activation or inhibition depending upon whether the cellular receptors to which they are fixed are FcγRII/III (activation) or FcγRIIb (inhibition)^56–58^. Thus, the rise of IgG1 and IgG2a observed in allergenized mice that did not decline after treatment with a vaccine or placebo may be either pathogenic or may reflect attempts by the immune system to counteract the allergic manifestations.

Allergic mice in all vaccine-treated groups had profound changes in respiratory tissues. All had significant reduction in lung inflammation, a lesser degree of tissue remodeling, and less expression of Th2 cytokine genes (*IL-4*, *IL-5*, and *IL-13*) and *TNF-α* compared to placebo mice. However, only mice treated with L-T289-Per a 9 and L-T167-Per a 9 had significant upregulation of immunosuppressive cytokine gene expressions (*TGF-β*, *IL-10*, and *IL-35* for L-T289-Per a 9, and *TGF-β* and *IL-10* for L-T167-Per a 9), which implied that these vaccines could cause immune deviation in allergic mice from an overexuberant Th2 response to a regulatory response (i.e., generation of effective Tregs, which is a signature of successful AIT). The mechanisms of Tregs relative to control of allergic processes via suppression of pathogenic effector cells, including innate lymphoid cell 2 (ILC2), mast cells, basophils, DCs, and effector Th2, Th1 and Th17, have been studied extensively^59–62^. The mechanisms may be direct by releasing suppressive factors and cell-cell contact, or indirect via modulation of APCs. Importantly, Tregs can release immunosuppressive cytokines (*IL-10*, *TGF-β*, and *IL-35*)^63^, granzymes, and perforin^64,65^ that antagonized the effector cell activities and kill them. Tregs generate adenosine from ATP using cell surface ectonucleotidases (CD39 and CD73), and transfer cAMP to both CD4^+^ and CD8^+^ Teff, which causes suppression of TCR-induced Ca^2^+, NF-AT, and NF-κB signaling and IL-2 production, which effectuates Teff anergy as well as the induction of pTregs^66–68^. Tregs downregulate costimulatory molecules on APCs via CTLA-4 (CD152), impede antigen-presentation via LAG-3, and enhance formation of aggregate around immature DCs by LFA-1, which blocks allergen-specific Teff responses^59,60^. Tregs modulate sulfur-based redox metabolism by blocking GSH redistribution from the nucleus to the cytoplasm in target cells, which leads to suppression of cell activation and proliferation^69^. Moreover, interaction of Treg CTLA-4 and APC CD80/86 promotes generation of IDO which is a heme-containing enzyme that catalyzes degradation of tryptophan to kynurenine, leading to starvation of effector T cells and direct cell cycle arrest and causes generation of inducible Tregs which suppress the immune response^70–72^. Increment of IDO1 together with up-regulation of the immunosuppressive cytokine genes in lung tissues of allergic mice treated with L-T289-Per a 9 and L-T167-Per a 9 indicate that the regulatory immune response, which is the most required immunologic feature of AIT, could be effectively induced by the liposome-entrapped Tregitope-Per a 9 vaccines.

Liposome-entrapped recombinant Per a 9 did not upregulate immunosuppressive cytokines and IDO1 increment in the lungs of vaccine-treated allergic mice in this study, implying that the vaccine could not induce regulatory lymphocyte generation. Our previous study demonstrated that liposome-entrapped native Per a 9 vaccine could induce upregulation of *IL-10* in lung tissue of treated CR-allergic mice^28^. This difference in findings may be due to that native Per a 9 purified from *P*. *americana* extract in the previous study contained many isoforms^36^, and one isoform may have contained peptide epitope(s) for lung Tregs that was/were lacking in the monoisoform of the recombinant Per a 9. Nevertheless, allergic mice treated with the L-recombinant Per a 9 vaccine in this study had significant reduction in Th2 and pro-inflammatory cytokine gene mRNAs, which correlated with reduction in lung inflammation, indicating that the vaccine had therapeutic efficacy. L-Per a 9-treated mice had upregulation of the *IFN-γ* gene, which is the signature of Th1 response. The level of endotoxin in the *E*. *coli* derived-Per a 9 was 0.25 EU/µg. Each mouse received 20 µl (50 µg) of L-Per a 9/dose which contained 12.5 EU of the endotoxin (the L-T289-Per a 9 and L-T167-Per a 9 contained 10 and 11.5 EU/dose, respectively, – they did not induce IFN-γ response). This amount of endotoxin should be released slowly from the liposome and should not induce IFN-γ response of cells in the mouse respiratory tract^73^. Spleen cells of BALB/c mice injected with DH5α *E*. *coli* endotoxin at 5, 8.5, and 12.5 EU were all found not to express any of the TNF-α, IL-6, IFN-γ and IL-2 genes at 2, 12 and 24 hours post-injection^74^. Thus, the *IFN-γ* expression in lungs of mice treated with L-Per a 9 should not be due to the endotoxin contained in the vaccine. The Th1 *IFN-γ* has been shown to be crucial for resolution of allergy-related immunopathologies by various mechanisms^75^, including regulation of allergen presentation to T lymphocytes^76^, differentiation of naïve T cells toward Th1 phenotype and/or inhibition of Th2 cell recruitment/differentiation^77,78^, suppression of Th2 cytokine production from activated T cells^79^, inhibition of effector cell recruitment to the site of inflammation^80^, induction of apoptosis of Th2 cells and eosinophils^81^, blockage of IgE isotype switch in B cells^82^, induction of IDO1 expression^72^ and induction of nitric oxide (NO) production^83^. The airway smooth muscle cell hyperplasia and hypertrophy observed in CR-allergic mice contributed to airway dysfunction. NO production induced by the Th1 cytokine can inhibit proliferation and DNA synthesis in airway smooth muscle cells^83^, which should lead to a reversal in tissue remodeling. Taken together, the efficacy of the L-recombinant Per a 9 vaccine for mitigating allergic features in treated mice was likely due to the negative regulatory role and effect of Th1 cytokine on allergic manifestations^75^.

In summary, mice that had signatures of allergy to *P*. *americana* crude extract were generated. They were treated with one of three liposome-encapsulated vaccines made of mouse Tregitope289-Per a 9, Tregitope167-Per a 9, or recombinant Per a 9 alone – all compared to placebo. All vaccinated allergic mice had significant reduction in Th2, proinflammatory cytokine expressions, and lung inflammation, with reversal of respiratory tissue remodeling. However, only liposome-encapsulated T289-Per a 9 and T167-Per a 9 induced the expression of immunosuppressive cytokine genes (*IL-10*, *TGF-β*, and *IL-35* for L-T289-Per a 9, and *IL-10* and *TGF-β* for L-T167-Per a 9) and increment of IDO1, which indicated that the mechanisms of allergic disease suppression and the reversal of tissue remodeling induced by these two vaccines occurred via generation of regulatory lymphocytes. Liposome-entrapped-recombinant-Per a 9 did not cause upregulation of immunosuppressive cytokines and increase of IDO1, but instead induced high expressions of Th1 cytokine in lung tissue of vaccine-treated allergic mice. The findings from this study provide compelling evidence that both liposome-encapsulated vaccines made of single refined major allergen alone and single refined major allergen linked with Tregitopes is effective for reducing respiratory tissue inflammation and microstructural impairment caused by the allergen, but through different mechanisms.

## Electronic supplementary material

Supplementary Information
