# Supplementary material for: Tregitope-linked Refined Allergen Vaccines for Immunotherapy in Cockroach Allergy
Source: Sci Rep. 2018 Oct 19;8:15480. doi: 10.1038/s41598-018-33680-9 (PMC6195530; doi:10.1038/s41598-018-33680-9)

Tregitope-linked Refined Allergen Vaccines for Immunotherapy in Cockroach Allergy

Pannathee Prangtaworn[1,2], Urai Chaisri[3], Watee Seesuay[2], Kodchakorn Mahasongkram[2], Nattawat Onlamoon[4], Onrapak Reamtong[5], Anchalee Tungtrongchitr[2], Nitaya Indrawattana[6], Wanpen Chaicumpa[2] & Nitat Sookrung[2,4]

[1]Graduate Program in Immunology, Department of Immunology, Faculty of Medicine Siriraj Hospital, Mahidol University, Bangkok, Thailand. [2]Center of Research Excellence on Therapeutic Proteins and Antibody Engineering, Department of Parasitology, Faculty of Medicine Siriraj Hospital, Mahidol University, Bangkok, Thailand. [3]Department of Tropical Pathology, Faculty of Tropical Medicine, Mahidol University, Bangkok, Thailand. [4]Biomedical Research Incubator Unit, Department of Research, Faculty of Medicine Siriraj Hospital, Mahidol University, Bangkok, Thailand. [5]Department of Tropical Molecular Biology and Genetics, Faculty of Tropical Medicine, Mahidol University, Bangkok, Thailand. [6]Department of Microbiology and Immunology, Faculty of Tropical Medicine, Mahidol University, Bangkok, Thailand. Correspondence and request for materials should be addressed to N.S. (email: nitat.soo@mahidol.ac.th)

Supplementary Table 1. Oligonucleotide primers used in qRT-PCR for monitoring cytokine gene expressions.

**Gene**	**Primer: sequence**	**Size of PCR product (bp)**
IL-4	F: 5′-TCGGCATTTTGAACGAGGTC-3′<br>R: 5′-GAAAAGCCCGAAAGAGTCTC-3′	216
IL-5	F: 5′-ATGATCGTGCCTCTGTGCCTGGAGC-3′<br>R: 5′-CTGTTTTTCCTGGAGTAAACTGGGG-3′	242
IL-13	F: 5′-CGCTGGCGGGTTCTGTGTAG-3′<br>R:5′-GAGGCTGGAGACCGTAGTGGG-3′	127
TNF-α	F: 5′-CATCTTCTCAAAATTCGAGTGACAA-3′<br>R: 5′-TGGGAGTAGACAAGGTACAACCC-3′	175
IL-12a (p35)	F: 5′-CCACCCTTGCCCTCCTAAAC-3′<br>R: 5′-GTTTTTCTCTGGCCGTCTTCA-3′	132
IL-12b (p40)	F: 5′-GGAAGCACGGCAGCAGAATA-3′<br>R: 5′-AACTTGAGGGAGAAGTAGGAATGG-3′	180
IL-17A	F: 5′-CAGGGAGAGCTTCATCTGTGT-3′<br>R: 5′-GCTGAGCTTTGAGGGATGAT-3′	94
IFN-γ	F: 5′-AACGCTACACACTGCATCTTGG-3′<br>R: 5′-GACTTCAAAGAGTCTGAGG-3′	237
IL-10	F: 5′-CGGGAAGACAATAACTG-3′<br>R: 5′-CATTTCCGATAAGGCTTGG-3′	186
TGF-β	F: 5′-CAAGGGCTACCATGCCAACT-3′<br>R: 5′-AGGGCAAGGACCTTGCTG-3′	84
IL-35 (ebi3)	F: 5′-CAATGCCATGCTTCTCGGTAT-3′<br>R: 5′-GGACGTGGATCTGGTGGAGTT-3′	84
β-actin	F: 5′-GGCCAACCGTGAAAAGATGA-3′<br>R: 5′-CACGCTCGGTCAGGATCTTC-3′	251

Supplementary Table 2. Grades and histological features of mouse lung sections after staining with (A) hematoxylin and Eosin (H&E) dyes, (B) Periodic Acid-Schiff and (C) Mannson's Trichrome stain.

A

Grade of lung histopathology	Histological features revealed by H & E staining
0	Bronchiole lined with single layer of ciliated columnar epithelial cells (large bronchiole) or cuboidal epithelial cells (small bronchiole). Submucosa contained single layer of smooth muscle cells with thin layer of connective tissue. Capillaries were lined with single layer of squamous epithelium and no inflammatory cell infiltration into peribronchiolar tissue
1	Thickened bronchiole which lined with ciliated pseudo-stratified columnar epithelial cells; mild degeneration of smooth muscle cells; alveoli showed slightly thickened wall with few inflammatory cells in the interstitial area
2	Hyperplasia of bronchiolar epithelial cells; moderate degeneration of submucosal smooth muscle cells; more inflammatory cell infiltrated into peribronchiolar area than grade 1
3	Hyperplasia of bronchiolar epithelial cells; detachment and degenerative change of the submucosal smooth muscle cells; infiltration of inflammatory cells which forming 2-3 cell layers around bronchioles
4	Bronchiolar epithelial cell hyperplasia and occlusion of the airway by sloughed-off necrotic epithelial cells; intense inflammatory cell infiltration which formed more than 4 layers around bronchioles

B

Grade of lung histopathology	Histological features revealed by PAS staining
1	Less than 25% of airway epithelium were stained
2	About 26–50% of airway epithelium were stained
3	About 51–75% of airway epithelium were stained
4	More than 75% of the airway epithelium were stained

C

Grade of lung histopathology	Histological features revealed by Mannson's Trichrome Staining
0 (normal)	Normal collagen content of alveolar septa; thin-walled alveoli
1	Slight deposition of collagen in the alveolar septa with mild fibrotic change
2	Mild collagen deposition and thick fibrosis of alveolar septa
3	Moderate collagen deposition; continuous fibrosis of the alveolar septa
4	Excessive collagen deposition leading to thickened alveolar septa, compressed alveoli, confluent fibrotic masses, and fibroplasia

Supplementary Figure 1. Flow diagram for preparing Tregitopes.

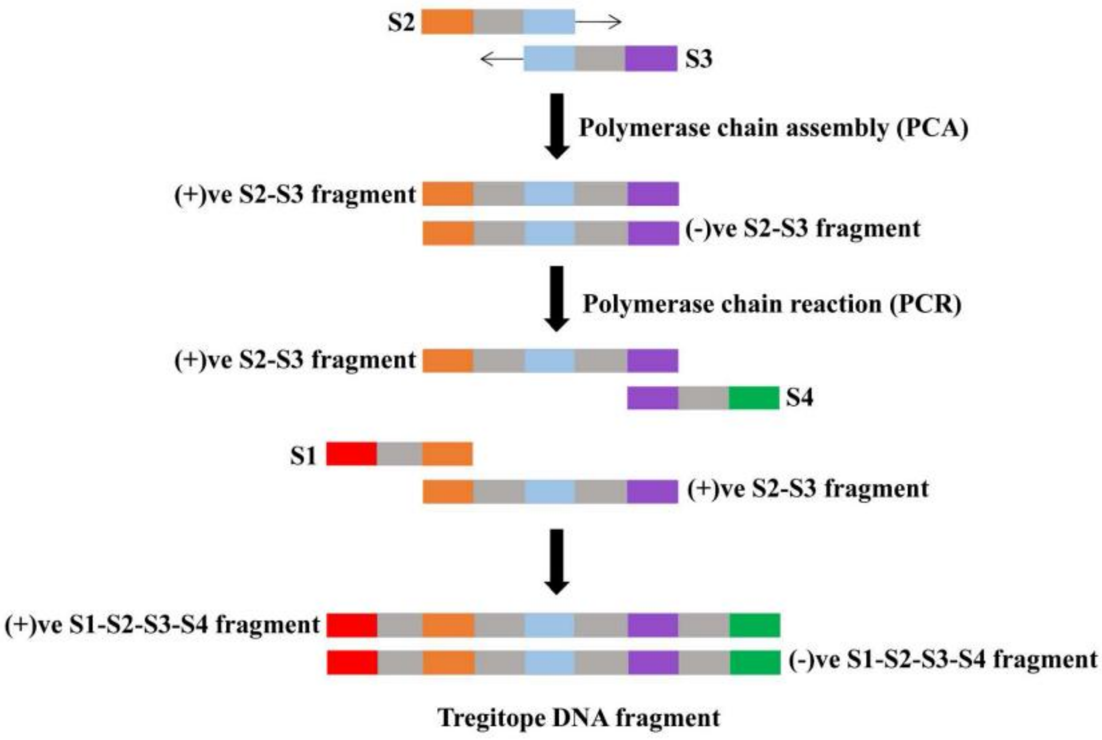

Supplementary Figure 2. The purified recombinant Per a 9, T289-Per a 9 and T167-Per a9 proteins after SDS-PAGE and CBB staining (**A**) and their respective Western blot patterns (**B**).

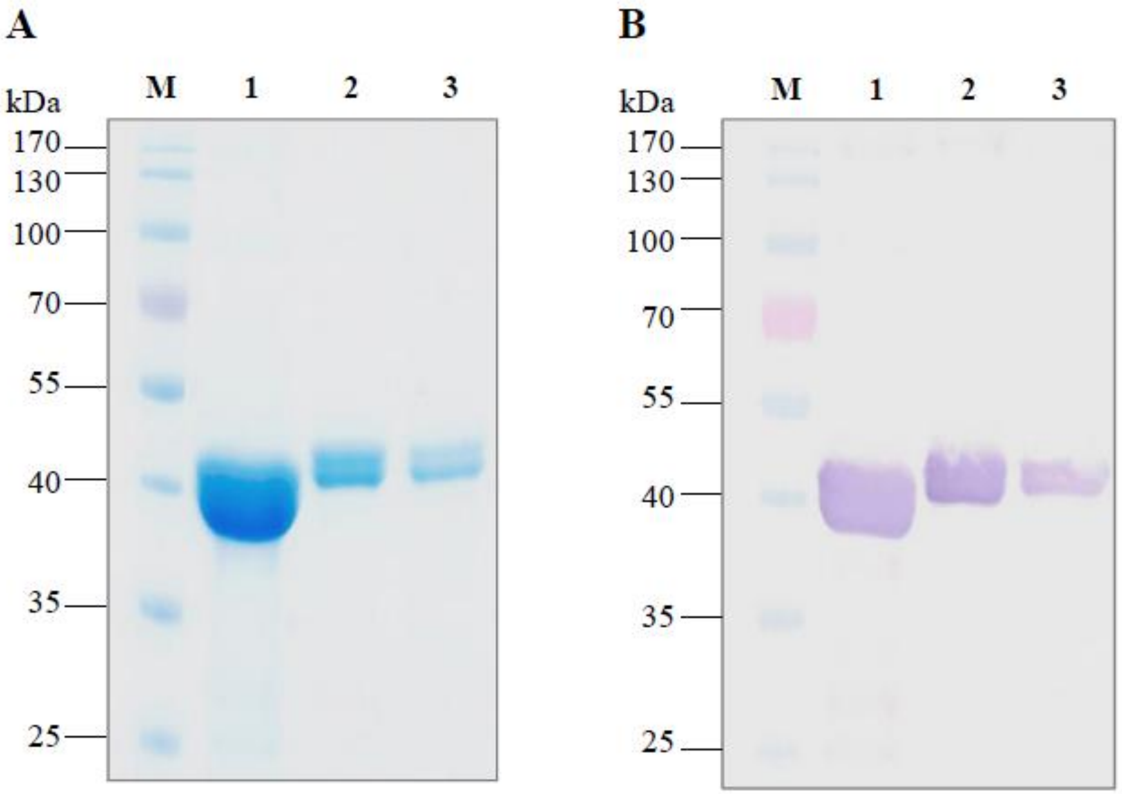

Supplementary Figure 3. Levels of serum-specific IgE (**A**), IgG1 (**B**), and IgG2 (**C**) in allergic mice treated with one of the three vaccines (L-T289-Per a 9, L-T167-Per a 9 or L-Per a 9) or placebo (L-P).

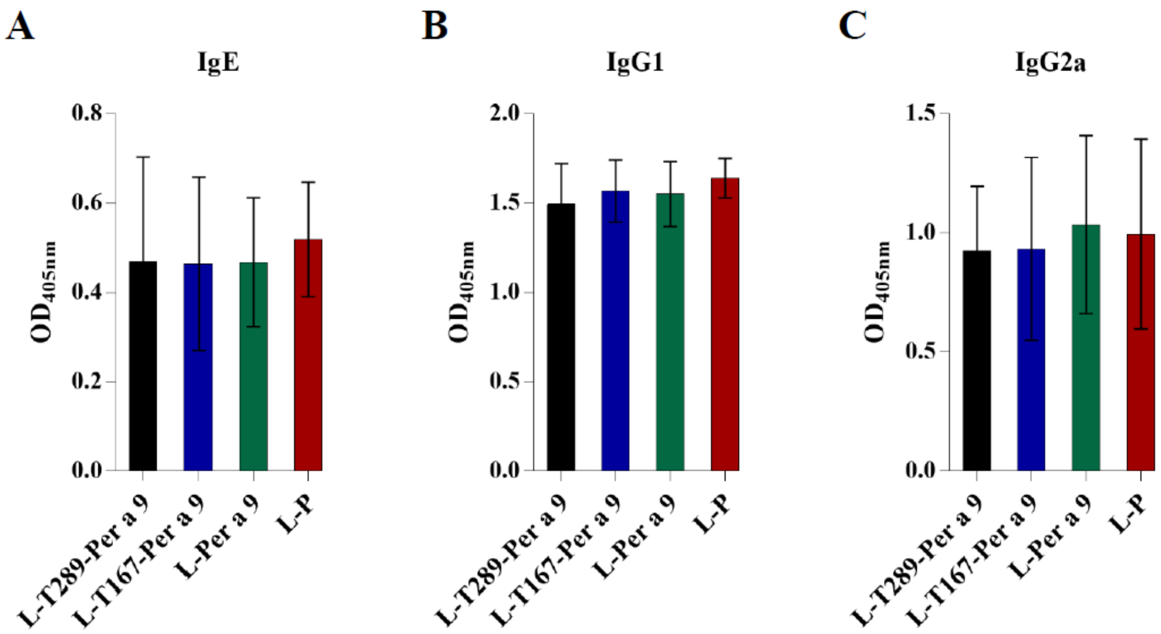

Supplement: Supplementary file 1 — Supplementary Information [file 41598_2018_33680_MOESM1_ESM.pdf]
